# Evaluation of phytochemical content, antimicrobial, cytotoxic and antitumor activities of extract from *Rumex hastatus* D. Don roots

**DOI:** 10.1186/s12906-015-0736-y

**Published:** 2015-07-03

**Authors:** Sumaira Sahreen, Muhammad Rashid Khan, Rahmat Ali Khan, Taibi Ben Hadda

**Affiliations:** Botanical Sciences Division, Pakistan Museum of Natural History, Garden Avenue, Shakarparian, Islamabad, Pakistan; Department of Biochemistry, Faculty of Biological Sciences, Quaid-i-Azam University Islamabad, Islamabad, Pakistan; Department of Biotechnology, Faculty of Biological Sciences, University of Science and Technology Bannu, Khyber Pakhtunkhwa, Pakistan; Department of Chemistry Mohammed First University, Faculty of Sciences Materials Chemistry Laboratory, Oujda, 60000 Morocco

**Keywords:** *Rumex hastatus*, Roots, Phytochemicals, Antimicrobial, Cytotoxic, Antitumor

## Abstract

**Background:**

Being a part of Chinese as well as ayurdic herbal system, roots of *Rumex hastatus* D. Don (RH) is highly medicinal, used to regulated blood pressure. It is also reported that the plant is diuretic, laxative, tonic, used against microbial skin diseases, bilious complaints and jaundice. The present study is conducted to evaluate phytochemical, antimicrobial, antitumor and cytotoxic activities of extract obtained from *R. hastatus* roots.

**Methods:**

RH roots were powdered and extracted with methanol to get crude extract. Crude extract was further fractioned on the basis of increasing polarity, with n-hexane (HRR), chloroform (CRR), ethyl acetate (ERR), n-butanol (BRR) and residual aqueous fraction (ARR). Methanol extract and its derived fractions were subjected to phytochemical screening and assayed for antibacterial activities via agar well diffusion method. Antifungal activities were checked through agar tube dilution method whereas potato disc assay was employed for the determination of antitumor activity. On the other hand cytotoxic activities were conducted using brine shrimps procedures.

**Results:**

The results obtained from phytochemical analysis indicate the presence of alkaloids, anthraquinones, flavonoids and saponins in all the fractions. Most of the plant fractions showed substantial antimicrobial activities, which is in accordance with the spacious use of tested plant samples in primary healthcare center. Fractions of *R. hastatus* roots for cytotoxicity were tested as an effective cytotoxic was found as BRR > MRR > CRR > ARR > ERR > HRR. Ranking order of fractions of *R. hastatus* roots for effective antitumor screening was found as MRR > BRR > ARR > CRR > ERR > HRR.

**Conclusions:**

These results showed that *R. hastatus* appeared as an important source for the discovery of new antimicrobial drugs and antitumor agents; verify its traditional uses and its exploitation as therapeutic agent.

## Background

Several countries use plants as a best source of medicine to treat infections and other disorders and nowadays most potent and powerful drugs are derived from plants [1]. Natural products derived from plant extracts/fractions are novel therapeutic agents for various infectious as well as degenerative diseases. In herbal medicines various parts of the plant (root, stem, flower, fruit, twigs exudates and modified plant organs) are used having diverse therapeutic properties. To utilize these plants, collected on minute scale by local communities and folk healers, while to trade for herbal industries numerous other plants are collected in large amount as a raw material [2]. People of developed and developing countries have turned back their attention towards botanicals for a substitutive health care services as they are an accessible and economical source in comparison to synthetic medicines. In recent years for the management and protection against pathogens, a large number of plants have been examined for their antimicrobial characteristics as an integrative system of medicine. For the treatment of human pathogenic diseases, use of plant extracts or fractions having antimicrobial properties can be of great importance. Natural biological active compounds in plants have a significant role in plants defense mechanism and also important for their unambiguous physiological action in human body. Because of the therapeutic property, secondary metabolites (flavonoids, alkaloids, tannins, saponins, and terpenoids) are becoming a part of the integrative health care system as supportive and alternative medicines [3]. For the cure of infectious diseases the unsystematic use of commercial antimicrobial medicines has led to development of numerous drug resistances in human pathogenic microorganisms. Besides, a number of side effects like hypersensitivity, allergic reactions and immune suppression are rarely associated with the antibiotics. Further, food preservation requires evaluation of natural resources such as herbal fractions and isolates with antimicrobial properties as the long historic use of herbs has proved their safety and efficacy in various traditional medicine systems. Recent trends for the use of natural remedies as antimicrobial have increased their use in food, cosmetic and pharmaceutical products which have been screened *in vitro* and indicated antimicrobial and other diverse properties [4]. Novel compounds have a vast therapeutic ability, lessen various adverse effects, and are often coupled with synthetic antimicrobials. One of the valuable means used to screen bioactive compounds from plant extracts is brine shrimp lethality assay [5]. It is considered as a convenient probe for primary evaluation of toxicity [6]. Medicinal plants are good source to obtain a wide range of drugs in view of the fact that, a single plant can be used to treat more than one ailment. *Rumex hastatus* D. Don, belongs to the Polygonaceae family. The plant is suffrutescent richly branching shrub. It grows up to 90 to 120 cm tall and leaves with petioles of the same length as the blade; blade hastate, panicles terminal with erect-divergent, mostly simple branches, nut up to 2 mm long, brown, and long spindle-shaped roots. It is distributed in northern Pakistan, north east Afghanistan and south west of China, growing between 700 to 2500 m, sometimes grows as pure population [7]. The present study is therefore arranged to screen and evaluate antibacterial, antifungal, antitumor and cytotoxic activities as well as phytochemical analysis of crude methanol extract.

## Methods

### Extract preparation

*R. hastatus* District roots were collected from Havelian, Abbottabad, Pakistan. The plant specimen are documented by their native names and then authenticated by Prof. Dr. Mir Ajab Khan, Department of Plant Sciences, Quaid-i-Azam University, Islamabad. Voucher specimens with Accession No. 27813 *(R. hastatus)* were placed at the Herbarium of Pakistan. Shade dried roots were powdered in a Willy Mill to 60-mesh size and used for solvent extraction. Five kilogram of each powdered sample was extracted twice with 10 L of 95 % methanol at 25 °C for 48 h. For filtration Whatman No. 1 filter paper was used and then the filtrate was concentrated on rotary evaporator (Panchun Scientific Co., Kaohsiung, Taiwan) under reduced pressure at 40 °C. In order to resolve the compounds with escalating polarity, a part of the extract was suspended in distilled water and partitioned with n-hexane (HRR), chloroform (CRR), ethyl acetate (ERR), n-butanol (BRR) and residual aqueous fraction (ARR).

### Determination of the phytochemical constituents

The extract of *R. hastatus* roots and its derived fractions were evaluated for the occurrence of alkaloids, saponins and terpenoids [8], whereas anthraquinone, phlobatannins and cardiac glycosides [9], as well as flavonoids and tannins [10], using standard phytochemical methods.

### Antimicrobial activities

All the microbes obtained from Department of Microbiology QAU, Islamabad, Pakistan while antibiotics were obtained from GlaxoSmithKline however the thymol and camptothecin are purchased from Sigma.

### Antibacterial activity

For antibacterial activity the strains used were *Bacillus subtilis* (ATCC 6633), *Enterobactor aerogenes* (ATCC 13048), *Escherichia coli* (ATCC 15224), *Klebsiella pneumoniae* (MTCC 618), *Micrococcus luteus* (ATCC 10240), *Pseudomonas aeroginosa* (ATCC 27853), *Salmonella typhy* (ATCC 0650), and *Staphylococcus aureus* (ATCC6538). Antibacterial activity of plant extract and its derived fractions was investigated by agar well diffusion method [11] using nutrient agar medium. Extract and fractions of different concentration were prepared in DMSO. Erythromycin and nalidixic acid were used as positive controls whereas DMSO was used as a negative control. The lowest concentration inhibiting growth was taken as the minimum inhibitory concentration (MIC).

### Antifungal activity

The agar tube dilution method was used for antifungal activity of plant extracts was determined according to the protocol reported by Duraipandiyan and Ignacimuthu [12] using *Aspergillus flavus (0064)*, *Aspergillus fumigatus (66), Aspergillus niger (0198)* a*nd Fusarium solani (0300).* Tubes were prepared in triplicate for each fungus species. Percentage inhibition of fungal growth for each concentration of fractions was determined by the following formula;

Percentage inhibition of fungal growth = (100 - linear growth in test (mm) / linear growth in control (mm) × 100

### Cytotoxic brine shrimp assay

*R. hastatus* and its various fractions were analyzed by using brine shrimps hatched in saline [13]1 10, 100, 1000 ppm was incubated with brine shrimps. After 24 h of incubation survivors were counted with help of 3× magnifying glass and calculation was done using Abbot^’^s formula;

% Death _=_ (Sample-control/control) × 100

### Antitumor potato disc assay

*R. hastatus* and its derived fractions were analyzed for their antitumor activity by antitumor potato disc assay reported by Ferrigini *et al.,* [14]. Bacterial culture of *Agrobacterium tumefaciens* (At 10) was used. Number of tumors per disc was counted and percentage inhibition for each concentration was determined as follows:

% age inhibition = 100 - [(average number of tumors of sample) / (Average number of tumors of negative control)] × 100

20 % tumor inhibition was considered significant.

### Statistical analysis

Data of *in vitro* assays recorded were analyzed with help computerized Graph prism pad software to determined standard error, IC_50_ and LD_50_.

## Results and discussion

Plants and plant derived fractions are used for medication since prehistory. As reported by World Health Organization (WHO) about 80 % of the world’s population depends mainly on conventional medicines that involve the use of plant extracts [15, 16]. The folkloric use of *R. hastatus* for treating infections may be justified by the charisma of anthraquinones as they possess antimicrobial [17] and antiparasitic properties [18]. Coumarins are known as antioxidant, anti-inflammatory, antiviral, anticarcinogenic and hepatoprotective agent like hydroxycoumarins have ability to chelate metal ions and scavenge free radicals [19]. Present study supports the examined plant parts as large sources of bioactive chemicals specifically with reference to alkaloids, anthraquinones, coumarins, flavonoids, tannins and saponins that ought to be isolated and monitored for biological activities as reported in traditional and therapeutic utilization [19].

In the present study *R. hastatus* roots showed alkaloids, anthraquinones, flavonoids and saponins were present in MRR and its various fractions such as HRR, ERR, CRR, BRR and ARR (Table 1). Presence of tannins was not detected in HRR and ARR while terpenoids were remained absent in HRR and ERR. Cardiac glycosides were present in MRR, ERR and BRR while coumarins were not reported in ERR and ARR. Analysis of phlobatannins revealed their presence only in MRR and BRR.

**Table 1:** Phytochemical constituent of *R. hastatus* methanol extract and its fractions

Phytochemical constituents						
	MRR	HRR	ERR	CRR	BRR	ARR
Alkaloids	+	+	+	+	+	+
Anthraquinones	+	+	+	+	+	+
Cardiac glycosides	+	-	+	-	+	-
Coumarins	+	+	-	+	+	-
Flavonoids	+	+	+	+	+	+
Phlobatannins	+	-	-	-	+	-
Saponins	+	+	+	+	+	+
Tannins	+	-	+	+	+	-
Terpenoids	+	-	-	+	+	+

+, present; −, absent

MRR; *R. hastatus* methanol extract, HRR; *R. hastatus* n-hexane fraction, CRR

*R. hastatus* chloroform fraction, ERR; *R. hastatus* ethyl acetate fraction, BRR

*R. hastatus* n-butanol fraction,ARR; *R. hastatus* residual aqueous fraction

In the present study different bioassays were used to characterize the plant fractions. The MIC measurement is a quantitative method, corresponds to the lowest applied amount of test material able to inhibit any visible microbial growth. According to Lambert and Pearson [20], MIC is a standard measure documented for the susceptibility of organisms to inhibitors in biological assays. The antibacterial activity was tested against both gram-negative bacteria as well as gram-positive bacteria. Table 2 describes the antibacterial activity as MIC value of various fractions of plant samples against tested bacteria. The results obtained from the present study for all tested bacteria ranged from 0.1 to 10 mg/ml. Gram-positive bacteria such as *Staphylococcus aureus* was inhibited by MIC value (1 mg/ml) of MRR; 2.5 mg/ml of HRR, BRR; 5 mg/ml of CRR while rest of the fractions didn’t inhibit the growth of *Staphylococcus aureus*. On the other hand, *Bacillus subtilis* was inhibited by ERR (0.1 mg/ml); MRR (1 mg/ml); BRR (0.5 mg/ml); ARR (2.5 mg/ml); CRR (5 mg/ml) while remaining fractions did not inhibit the growth of the respective bacteria (Table 2). In case of gram negative bacteria *Klebsiella pneumoniae*‘s growth was inhibited by MRR (5 mg/ml); HRR, ERR (10 mg/ml) while remaining fractions did not inhibit the growth of the respective bacteria. *Pseudomonas aeroginosa* inhibited the growth at 0.1 mg/ml. Growth of *Salmonella typhy* was inhibited by HRR (0.5 mg/ml); BRR (0.25 mg/ml); while rest of the fractions inhibited the growth at 0.1 mg/ml. However, BRR inhibited the growth of *Enterobacter aerogenes* with MIC (1 mg/ml); CRR, MRR (2.5 mg/ml); ARR (5 mg/ml); while remaining fractions did not show inhibition of the concerned bacteria (Table 2). MIC against *Micrococcus luteus* and *Escherichia coli* had no value, showing no antibacterial activity of any of the fractions. The activity against both type of bacteria (gram-positive and gram-negative bacteria) indicate that fractions contain broad spectrum of antibiotic compounds or metabolic toxins. These fractions may possibly be functional for the strengthening of new antimicrobial drugs. Numbers of authors have focused on antibacterial and antifungal potency of flavonoids [21, 22]. Antimicrobial activity may be attributed to plant bioactive compounds to make complex with bacterial cell wall [17] and thus inhibiting the microbial growth.

**Table 2:** The Minimum inhibitory concentration (mg/ml) of *R. hastatus*

Bacteria								
	MRR	HRR	ERR	CRR	BRR	ARR	Erythromycin	Nalidixic acid
*B. subtilis*	1.0 ± 0.01	-	0.1 ± 0.01	5.0 ± 0.5	0.5 ± 0.01	2.5 ± 0.07	0.006 ± 0.0001	0.001 ± 0.0007
*E. aerogenes*	2.5 ± 0.02	-	-	2.5 ± 0.3	1.0 ± 0.01	5.0 ± 0.3	0.007 ± 0.0002	0.002 ± 0.001
*E. coli*	-	-	-	-	-	-	0.009 ± 0.0002	0.07 ± 0.009
*K. pneumoniae*	5.0 ± 0.3	10.0 ± 0.2	10.0 ± 0.9	-	-	-	0.004 ± 0.0002	0.001 ± 0.0004
*M. luteus*	-	-	-	-	-	-	0.006 ± 0.0001	0.001 ± 0.0003
*S. typhy*	0.1 ± 0.001	0.5 ± 0.01	0.1 ± 0.01	0.1 ± 0.01	0.25 ± 0.1	0.1 ± 0.01	0.001 ± 0.0002	0.007 ± 0.0008
*P. aeroginosa*	0.1 ± 0.002	0.1 ± 0.01	0.1 ± 0.07	0.1 ± 0.02	0.1 ± 0.01	0.1 ± 0.01	0.003 ± 0.0001	0.001 ± 0.0001
*S. aureus*	1.0 ± 0.07	2.5 ± 0.1	-	5.0 ± 0.6	2.5 ± 0.01	-	0.007 ± 0.0002	0.001 ± 0.0009

Mean ± SE (*n* = 3)

- = not active against tested microorganism

Our results suggest that antimicrobial activity of plant fractions doesn’t depends on only phenolics but other secondary metabolites are also involved. While, *R. hastatus* roots showed that inhibition against *A. niger* ranged from 20.4 ± 2.85 % to 68.3 ± 1.48 %, *A. flavus* at the range from 23.6 ± 1.32 % to 69.4 ± 3.18 %, *A. fumigatus* at a range from 15.5 ± 2.34 % to 51.3 ± 1.67 % and *F. solani* at a range from 14.1 ± 1.51 % to 34.9 ± 2.28 % (Table 3).

**Table 3:** Antifungal activity of the crude extract and various fractions of *R. hastatus* (Percent inhibition)

	MRR	HRR	ERR	CRR	BRR	ARR	Terbinafin
*A. niger*	39.1 ± 1.39	40.2 ± 2.83	48.9 ± 1.66	68.3 ± 1.48	28.4 ± 2.51	20.4 ± 2.85	84.5 ± 2.13
*F. solani*	25.4 ± 2.53	15.8 ± 1.39	14.1 ± 1.51	31.7 ± 2.49	31.7 ± 2.49	31.7 ± 2.49	81.78 ± 2.12
*A. flavus*	69.4 ± 3.18	36.4 ± 1.89	28.0 ± 1.34	36.0 ± 1.68	56.2 ± 2.77	23.6 ± 1.32	87.6 ± 2.70
*A. fumigatus*	22.6 ± 2.32	27.0 ± 2.56	43.2 ± 1.80	51.3 ± 1.67	15.5 ± 2.34	40.5 ± 1.07	88.4 ± 3.16

Mean ± SE (*n* = 3)

For the investigation of bioactive compounds, biological assays especially brine shrimp lethality assay (BSLA) is considered as necessary and suitable tools. According to Meyer *et al.* [6] who categorized crude extracts and pure compounds into toxic (LC50 value < 1000 ppm) and non-toxic (LC50 value > 1000 ppm), all tested showed good brine shrimp larvicidal activity excluding HRR. Moreover, Peteros and Uy [23] signified the presence of effective cytotoxic substances of plant extracts having LC50 values < 100 ppm to brine shrimp lethality.

Our findings were in strong accordance to that of Peteros and Uy [23] and suggest isolating new active compounds. *R. hastatus* roots for cytotoxicity were tested and their ranking order for effective cytotoxic was found as BRR > MRR > CRR > ARR > ERR > HRR (Table 4). About all of the tested samples showed their toxic effect at 1000 ppm apart HRR.). The larvicidal activity of *R. hastatus* were in consistent with the Hussain *et al.* [24] who reported the cytotoxic activity of methanol extracts of *Rumex* species and found that *R. hastatus* showed significant activity against brine shrimp larvae. It is considered that presence of a wide range of biologically active compounds with different structures and their synergistic effects may add to the overall activity of particular fraction.

**Table 4:** Illustration of % age mortality of brine shrimps at different concentrations of extract and fractions and respective LD50 values

Extracts				
	10 ppm	100 ppm	1000 ppm	LD50 (ppm)
MRR	1.76 ± 0.05	2.28 ± 0.17	3.65 ± 0.15	60.0 ± 3.5
HRR	1.05 ± 0.31	2.11 ± 0.02	2.18 ± 0.09	>1000
ERR	1.66 ± 0.05	1.50 ± 0.03	1.79 ± 0.06	705.5 ± 5.2
CRR	2.65 ± 0.22	2.13 ± 0.05	1.52 ± 0.12	65.7 ± 4.1
BRR	2.53 ± 0.31	2.81 ± 0.03	3.05 ± 0.09	15.4 ± 1.5
ARR	3.15 ± 0.08	3.16 ± 0.06	1.72 ± 0.23	100 ± 2.7
Thymol	1.62 ± 0.04	2.89 ± 0.07	2.75 ± 0.13	<10 ± 0.07

Mean ± SE (*n* = 3)

Antitumor potato disc is one of the best bioassay used for the detection of biologically active components in the botanical extracts [25]. Tumor inducing plasmid multiplies the plant’s cells excluding apoptosis phase and ultimately tumor is formed that has similarity with human and animal cancers, histology and nucleic acid [26, 27]. Several scientists have been used these methods over the past 15 years, and they appear to be adaptable to the purpose of standardization or quality control of bioactive compounds in such heterogeneous botanicals [28]. Table 5 describes that the ranking order for effective antitumor screening of fractions of *R. hastatus* roots was found as MRR > BRR > ARR > CRR > ERR > HRR. Different strains can be used to induce tumor on potato discs but strain Atl0 was found more prominent for producing tumor [29, 30] [29, 30]. Our results are in accord to other studies [31] who reported that tumor inhibition rates on potato discs are dependent on the concentration of the samples. Our findings confirm the previous reports of Islam *et al.* [30] and Ashraf *et al.* [32] who reported that the antitumor activity perhaps endorsed with the nature of biological active compounds and their strong solubility with appropriate solvent and also justifying the statement of Fatima *et al.* [31] that tumor induction was variable in case of different solvent extracts.

**Table 5:** % tumors inhibition of various fractions of *R. hastatus* against potato disc tumor

Extracts				
	10 ppm	100 ppm	1000 ppm	IC50 (ppm)
MRR	26.24 ± 1.20	69.33 ± 1.56	87.00 ± 0.85	60
HRR	19.43 ± 0.75	26.00 ± 0.52	37.47 ± 1.08	>1000
ERR	35.43 ± 0.38	47.62 ± 0.94	58.17 ± 1.20	340
CRR	32.47 ± 1.32	56.80 ± 2.17	75.82 ± 2.63	80
BRR	19.86 ± 0.44	64.36 ± 0.77	84.33 ± 1.43	70
ARR	24.65 ± 0.63	63.33 ± 2.43	78.33 ± 2.61	70
Camptothecin	49.2 ± 1.2	69.5 ± 2.3	88.9 ± 3.2	11

Mean ± SE (*n* = 3)

## Conclusions

The result of this study indicates the potential of *R. hastatus* roots as a source of therapeutic agent against microbial infection and tumor diseases.
